# Supplementary material for: Caregiver Survey-Based Perspectives on Digital Therapeutics for Children with Delayed Language Development
Source: Healthcare (Basel). 2025 Dec 15;13(24):3290. doi: 10.3390/healthcare13243290 (PMC12733230; doi:10.3390/healthcare13243290)
Supplement: Supplementary file 1 [file healthcare-13-03290-s001.zip › healthcare-4010008-supplementary.pdf]

Supplementary Table S1. Questionnaire Items by Domain

Domain	Number of questions (conditional question)	Item	Response Scale
Demographic characteristics	5	Childcare Experience, Relationship to the Child, Residence, Child's Gender, Birth Year and Month	Categorical (Single choice)
Characteristics	5(3)	Hearing Problems, Lingual Frenulum Surgery, Ankyloglossia (Tongue-tie), Disability Registration, Other Rehabilitation Therapies (Type of Disability Registration*, Disability Rating, Institution/Type of Rehabilitation Therapy)	Categorical (Single choice)<br>Categorical (Multiple choice*)<br>Conditional question<br>Open-ended option included
Status and Utilization of Speech Therapy	10	Referral Pathways for Suspected Language Delay*, Expected ST Outcomes*, Experience with Speech Therapy, Institution, Waiting Period for Therapy Initiation, Duration of Therapy, Frequency of Therapy Sessions, One-Way Travel Time (by Car), Maximum Tolerable Travel Time, Duration of Therapy Continuation	Categorical (Single choice)<br>Categorical (Multiple choice*)<br>Open-ended option included
Environment of App-Based Speech Therapy	6	Dual-Income Household, Daytime Caregiver for Child, Perceived Parental Role, Time Allowable, Available Home Space, Ownership of Tablet PC	Categorical (Single choice)<br>Conditional question<br>9-point Likert scale (Ordinal)
Preference for Digital Articulation Therapy via Applications	7(7)	Focus of App Use, Prerequisites for App Use*, Important Factors in App Selection & Use*, Experience with App-Based Education/Services, Intention to Use App, Preference Medial ST, Permissible Time (Satisfaction with App-Based Education/Services, Dissatisfaction with Application Use Experience, Reasons for Not Willing to Use, Preferred Content*, Gamified Content, Feedback Frequency, Willingness to Pay Amount)	Categorical (Single choice)<br>Categorical (Multiple choice*<br>Conditional question)<br>9-point Likert scale (Ordinal)<br>Open-ended option included
Total	33(10)		

Supplementary Table S2. Items based on the UTAUT2 model

UTAUT2	Question	Item
PE	Expected outcomes of Speech Therapy	Expected ST Outcomes
SI	Important factors influencing App selection and use	Important Factors In App Selection & Use
PV	Maximum willingness to pay	Willingness to Pay Amount
FC	Need for support in young children's app use at home<br>Maximum time caregivers could devote to their child's home-based speech therapy<br>Availability of a quiet space at home for speech therapy<br>Availability of a tablet PC at home for speech therapy	Perceived Parental Role<br>Time Allowable<br>Available Home Space<br>Ownership of Tablet PC
Use Intention	Intention to use the application while waiting for institutional therapy	Intention to Use App

Supplementary Table S3. Results of Multiple Regression Analysis

Variables	Unstd. Coeff. (B)	Std. coeff. (Beta)	Std. Error (SE)	p-value	VIF	Tolerance
Intercept	-.870		.608	.155		
Child's Gender → Intention	-.451	-.188	.179	.013	1.028	.973
Child's Age → Intention	.009	.012	.055	.872	1.06	.938
Child's ST experience → Intention	.442	.129	.289	.128	1.323	.756
PE → Intention	.381	.280	.107	<.001	1.146	.872
SI → Intention	.120	.047	.200	.551	1.153	.868
PV → Intention	.212	.189	.099	.034	1.437	.696
FC → Intention	-.175	-.143	.096	.071	1.137	.879
